# Evaluation of Sodium Bisulfate on Reducing *Salmonella* Heidelberg Biofilm and Colonization in Broiler Crops and Ceca

**DOI:** 10.3390/microorganisms9102047

**Published:** 2021-09-28

**Authors:** Megan Rose Pineda, James Allen Byrd, Kenneth James Genovese, Yuhua Zhang Farnell, Dan Zhao, Xi Wang, Allison Claire Milby, Morgan Brian Farnell

**Affiliations:** 1 Department of Poultry Science, Texas A&M University, College Station, TX 77843, USA; meganpineda25@gmail.com (M.R.P.); yfarnell@tamu.edu (Y.Z.F.); dz137@tamu.edu (D.Z.); wangxi@swun.edu.cn (X.W.); a.milby@tamu.edu (A.C.M.); 2 Southern Plains Agricultural Research Center, Agricultural Research Service, United States Department of Agriculture, College Station, TX 77845, USA; allen.byrd2@usda.gov (J.A.B.); kenneth.genovese@usda.gov (K.J.G.)

**Keywords:** *Salmonella*, water acidifier, biofilms, foodborne pathogens, meat safety, food-safety interventions, pathogen transmission, poultry, salmonellosis control, zoonoses

## Abstract

*Salmonella* Heidelberg (SH) on contaminated poultry causes economic and health risks to producers and consumers. We hypothesized that sodium bisulfate (SBS) would decrease SH biofilm on polyvinyl chloride (PVC) coupons and decrease the horizontal transfer of SH in broilers. Experiment 1: *Salmonella* Heidelberg biofilm was cultured with PVC coupons, which were treated with SBS at a pH of 3.5 for 10 min, 8 h, and 24 h. Experiment 2: Nine replicate pens per treatment were divided between two rooms. A seeder contact model was used to mimic a natural infection environment. Treatments consisted of tap water or sodium bisulfate in water at a pH of 3.5. *Salmonella* Heidelberg incidence and enumeration were measured in crops and ceca. Sodium bisulfate significantly reduced biofilm by 2.16 and 1.04 logs when treated for 8 and 24 h, respectively. Crop colonization was significantly decreased in trials 1 and 2 by 0.29 and 0.23 logs, respectively. Crop pH was significantly decreased in trial 2. Ceca colonization was significantly decreased in trial 1 by 0.39 logs. The results from the present study suggest that SBS may be administered to drinking water to decrease SH gut colonization and to reduce biofilm.

## 1. Introduction

Poultry are carriers of *Salmonella enterica* serotypes, which can cause salmonellosis in humans [1]. There are over 2500 *Salmonella* serotypes, with less than 100 that cause human disease [2]. The CDC reports drug-resistant *Salmonella* are a serious threat because of increased antibiotic resistant infections since 2009 [3]. Due to the concern of antimicrobial resistance, demand for antibiotic alternatives has increased. Antibiotic alternatives may include vaccines, probiotics, synbiotics, enzymes, organic/inorganic acids, phytobiotics, and prebiotics [4,5].

*Salmonella enterica* serovar Heidelberg (SH) is one of the top ten serovars associated with human disease isolated from poultry [6]. Egg contamination is a concern due to SH being one of a few *Salmonella* serotypes able to vertically transfer from hen to chick [7]. A surveillance study from 2002 to 2006 reported 96.6% of SH isolated came from poultry meat [8]. *Salmonella* Heidelberg has increasing resistance to common antibiotics and caused one of the largest multistate foodborne outbreaks from consumption of contaminated chicken [8,9]. Antimicrobial resistance continues to be an issue with SH, including multi-drug resistant (MDR) strains [7,10]. In humans, SH was MDR 30% of the time [11]. In 2014, 9.9% of isolated SH was MDR to five classes and 21.1% to three or more classes of antibiotics [12]. Due to the increased resistance combined with decreased usage of antibiotics, better alternatives are required.

The survival of microbial populations is increased by biofilm formation [13,14]. *Salmonella enterica* strains can form biofilms on abiotic surfaces, including ones found in all phases of poultry production [15,16]. Biofilm-forming abilities depend on growth conditions, contact surfaces, and serotypes or strains [17]. Attachment of biofilms to food contact surfaces (stainless steel, ceramics, glass, or plastic) can lead to cross-contamination of consumer products [18,19,20]. Reduction in *Salmonella* before processing is important to mitigate cross-contamination [21].

Government agencies regulate disinfectants and sanitizers, working together to standardize effective procedures. The U.S. Environmental Protection Agency (EPA) controls the efficacy, labeling, and handling of disinfectants [22]. Disinfection is a physical process, such as an ultraviolet light or chemical that kills microorganisms [23]. The Food and Drug Administration (FDA) and the US Department of Agriculture Food Safety and Inspection Service (FSIS) share mandates, regulating concentrations of commonly used disinfectants on surfaces that are commonly found in the production of meat, poultry, and eggs under guidance from the EPA [24]. Further control of pathogenic bacterial populations is mandated by USDA-FSIS to follow Hazard Analysis Critical Control Points (HACCP) [5]. In addition to disinfectants and sanitizers, inorganic acids also have some antimicrobial properties. Combinations of these strategies are used to reduce foodborne illness, but improvements are still needed. 

*Salmonella* control includes biosecurity throughout all phases of production. Controlled access, hygienic barriers, and pest control can all aid in limiting *Salmonella* [25]. However, even with enhance biosecurity measures, *Salmonella* remains an issue. Chemicals, such as acidified hypochlorite and peroxyacetic acid (PAA), used at the recommended FSIS and EPA ranges were ineffective against seven field strains of *Salmonella* biofilm [26]. Glutaraldehyde, hydrogen peroxide, and formaldehyde at a concentration of 1.0% (vol/vol) were unable to eradicate *Salmonella* on poultry house concrete floors [27]. Corcoran and colleagues found sodium hypochlorite (500 mg/L), sodium hydroxide (1 M), and benzalkonium chloride (0.02%) did not eliminate established 48 h or 168 h cultures of *Salmonella* Typhimurium or Enteritidis biofilm when treated for 90 min on concrete, glass, steel, polycarbonate, or tile coupons used to simulate food processing environments [28].

Clean drinking water is important for the health and performance of poultry. Biofilms in potable water systems may host pathogenic bacteria, which could be indicated by the presence of coliforms. There is no allowable level of coliform bacteria in drinking water, as it is an indicator of fecal contamination [22,23]. Maes and colleagues surveyed broiler farm microbial populations in outside water samples [29]. The total aerobic count ranged from 6 to 300 cfu/mL inside broiler houses [29]. Mixed-species biofilms can be made up of a combination of *Salmonella* and/or aerobic species [30]. Schaefer and colleagues reported *Salmonella* can readily colonize on silicone tubing as established mixed-species biofilms [30]. Broiler and layer farm water systems remain potential hot spots for *Salmonella* [27,31]. *Salmonella*-contaminated flocks also risk re-infection from contaminated drinkers [25].

Sodium bisulfate (SBS; NaHSO_4_), also known as sodium hydrogen sulfate, is categorized by the EPA as a mineral acid salt with antimicrobial properties that dissolves and releases a hydrogen ion, which decreases pH [32]. When used as a litter acidifier, SBS significantly decreased litter pH from 7.2 to 6.9, and reduced *Escherichia coli* (*E. coli*) by six logs after 2 weeks [33]. A lower litter pH can reduce bacteria that create ammonia gas from uric acid in excreta [34]. *Salmonella* Typhimurium (ST) was reduced by 1.3 logs in litter treated with SBS at a concentration of 100 lb/1000 ft^2^, which decreased litter pH from 8.3 to 3.5 [35]. Chicken drumsticks were inoculated with 10^8^ cfu/mL of SE and then treated for 0–3 days with SBS at concentrations of 1%, 2%, and 3% [36]. After 3 days, SBS significantly reduced pH from 7.42 to 1.64, 1.45, 1.31 and colonization by 0.92, 1.09, and 1.57 log cfu/g [36]. Micciche and colleagues found that at a pH of 1.21–1.54, SBS eliminated ST to 0 log cfu/mL in poultry, processing reused water in 5 min, which was a greater reduction than PAA, which reduced ST by 4–5 log cfu/mL at a pH of 4.02 [37]. When dog and cat food were treated with SBS at 0.2% and 0.4%, *Salmonella* Enteritidis was significantly decreased by 2 and 1.6 logs, respectively [38]. *Salmonella* Typhimurium was significantly decreased by 2.7 logs in rendered chicken fat (used for pet food products) by a 6-h SBS (0.5%) treatment [39]. Versatility of SBS in reducing *Salmonella* across platforms or mediums suggests its potential. 

Water lines can be an initial source of SH biofilm and minimizing colonization would provide cleaner drinkers. We hypothesized that SBS at a pH of 3.5 would eliminate SH biofilm on polyvinyl chloride (PVC) coupons and reduce the horizontal transfer of SH among broiler chicks. 

## 2. Materials and Methods

### 2.1. Bacterial Strains and Growth Conditions

Frozen stocks, maintained at –80 °C, of *Salmonella* Heidelberg (SH) were obtained from USDA-ARS (College Station, TX, USA). Cultures were passaged three times every 8 h in tryptic soy broth (TSB; Difco, Sparks, MD, USA) at 37 °C. Biofilm cultures were grown in Luria–Bertani broth (LB; HiMedia, Mumbai, India). All media were supplemented with novobiocin (25 µg/mL; Alfa Aesar, Haverhill, MA, USA) and nalidixic acid (LB^NN^; 20 µg/mL; MP Biomedicals, LLC, Illkirch, France) to control for extraneous bacteria. 

### 2.2. PVC Coupons

Coupons (PVC; 2 cm × 5 cm; 1” PVC Schedule 40) were cut with a rotary tool and lightly sanded. Coupons were soaked overnight in Alconox (White Plains, NY, USA), rinsed six times with tap water and one time in distilled water. The coupons were autoclaved for 15 min at 121 °C in water, and air dried overnight in a biosafety cabinet.

### 2.3. Sodium Bisulfate

Sodium bisulfate (SBS; Jones-Hamilton Co., Walbridge, OH, USA) was prepared in water via the manufacturer’s instructions. Briefly, 454 g of SBS was mixed into 16 L of water to create a stock solution. The stock solution was titrated into fresh tap water until a pH of 3.5 was obtained.

### 2.4. Biofilm Treatment

Biofilm formation on PVC was evaluated by using methods previously described [40,41]. Briefly, sterile coupons were initially inoculated in 1.0 × 10^9^ cfu/mL of SH suspension (bacterial attachment step) in 30 mL of LB^NN^ broth for 5 h under static conditions at 37 °C. Coupons were then removed using sterile forceps, rinsed with 1 mL of cold PBS to remove loose cells, and placed in a new tube with 30 mL of LB^NN^ for 6 days under static conditions (biofilm formation step) at 37 °C. The media were replaced every 48 h. Coupons were rinsed with 1 mL of cold phosphate-buffered saline (PBS) and air dried for 5 min in a HEPA-filtered biological safety cabinet during media replacement. All biofilm work was conducted in a biosafety cabinet. After 6 days, coupons were rinsed, dried, and placed in a treatment of 30 mL of SBS at a pH of 3.5 or sterile tap water for 10 min, 8 h, or 24 h at 37 °C.

### 2.5. Biofilm Analysis

Coupons were sonicated in 30 mL of PBS for 15 min at room temperature, using an ultrasonic cleaner (VWR, Radnor, PA, USA) at a fixed frequency of 35 kHz. Samples were serially diluted into PBS and directly plated onto XLT-4^NN^. Coupon rinsates were pre-enriched for 24 h in buffered peptone water (BPW; Difco, city, if any state, country), cultured into Rappaport–Vassiliadis broth (RV; Hardy Diagnostics, Santa Maria, CA, USA) and struck for incidence. All agar plates were incubated for 24 h at 37 °C. Values presented are the averages of six separate experiments on different days with triplicate coupon samples per treatment.

### 2.6. Animals and Handling Procedure

Day-of-hatch, male by-product broiler chicks were obtained from a commercial hatchery and placed on clean pine shavings in floor pens. The environment was climate controlled and age appropriate in disinfected animal biosecurity level 2 rooms, according to the primary breeder management guidelines [42]. Birds were monitored 2–3 times daily to check for morbidity, mortality, temperature, and relative humidity. Data loggers (CAS DataLoggers, Chesterland, OH, USA) measured the temperature and relative humidity every 5 min. Pen weights, feed, and water intake were measured across all trials to ensure consumption was consistent between treatments. Fresh tap water and SBS water were measured in a graduated cylinder daily. Feed was weighed back when the trial ended to calculate feed consumption. All birds were cared for under approved Texas A&M University Institutional Animal Care and Use Committee and Institutional Biosafety Committee protocols (IACUC 2019-0171; IBC 2019-073). Each trial was replicated twice at different time points.

### 2.7. Experimental Design and Treatment Groups

Chicks (*n* = 30/pen) were randomly placed across eighteen pens, sized 0.9 m by 1.5 m. Pens were assigned to one of two treatment groups with 9 pens (replicates). A balanced unmedicated starter ration and water were provided ad libitum that met or exceeded industry recommendations for nutrition. Upon arrival, a subset of ceca (*n* = 10) were collected for enrichment to verify chicks were *Salmonella* free. Ceca were macerated in BPW and incubated overnight at 37 °C. The following day, 0.1 mL of pre-enrichment was sub-cultured into RV at 37 °C overnight. The enrichment was struck for isolation onto XLT-4 without antibiotics to screen for wild-type strains. No *Salmonella* were detected.

Pre-seeder birds contaminated clean pine shavings to mimic a commercial broiler barn. Male broiler chicks were randomly selected and placed in groups of 30 chicks per pen. All pre-seeder chicks were orally gavaged with 0.5 mL of 2.0 × 10^7^ cfu/mL of SH upon arrival. On D7, all pre-seeder chicks were orally gavaged a second time with 0.5 mL of 2.0 × 10^8^ cfu/mL. Fecal grabs (*n* = 1/pen) were aseptically collected from the litter on D5 post-infection to confirm incidence of SH shedding into the environment. All pre-seeder birds were euthanized on D13 or D14 (based on hatchery schedule) by carbon dioxide (CO_2_) asphyxiation. Ceca (*n* = 10/pen) were collected for incidence.

New chicks were randomly placed in groups of 30 chicks per pen onto litter previously contaminated by pre-seeder chicks. Each pen included 10 seeder and 20 contact chicks. There were two treatments. One treatment received tap water. The second treatment received tap water treated with sodium bisulfate to a pH of 3.5. Each treatment pen was replicated nine times. Seeders were wing banded and orally gavaged with 0.5 mL of 2.0 × 10^8^ cfu/mL of SH to mimic horizontal transfer. On day 10, chicks were killed by CO_2_ asphyxiation. Ceca were removed from the seeders for incidence (*n* = 5/pen). Crop and ceca samples were aseptically removed from contact birds for enumeration and incidence (*n* = 10/pen). The crop contents were aseptically removed by clamping above and below the crop using Rochester-Carmalt forceps (VWR). Crop pH was measured (*n* = 5/pen) by diluting contents 10× in distilled water (Trial 1) or directly inserting a Hanna pH probe (Trial 2; Hanna Instruments, Smithfield, RI, USA).

### 2.8. Salmonella Challenge

*Salmonella* Heidelberg was harvested by centrifugation at 600 × *g* for 15 min at 4 °C to prepare the bird challenge. The pellet was resuspended in sterile cold PBS and washed twice prior to challenge. Optical density was measured spectrophotometrically at 625 nm at an absorbance value of 1.30 (SPECTRONIC^®^ 20+ SERIES Spectrophotometers, Thermo Fisher, Waltham, MA, USA) and estimated at 1.0 × 10^9^ cfu/mL, relative to an established standard curve. Concentration of the challenge stock was confirmed by serial dilution on xylose lysine tergitol-4 (XLT-4; Hardy Diagnostics, Santa Maria, CA, USA) agar with added supplement (Difco).

### 2.9. Bacteriological Analysis

Cecal contents were weighed, and approximately 0.25 g of the contents were serially diluted 1:10, 1:100, 1:1000, and 10,000 in PBS. Crop contents were weighed and stomached for 30 s in 5 mL of BPW (Stomacher). Crop samples were then serially diluted in PBS (1:10, 1:100, 1:1000, 1:10,000, 1:10,000). All enrichment samples were pre-cultured in BPW for 24 h before being sub-cultured into RV. All samples mentioned were cultured onto XLT-4^NN^ at 37 °C for 18–24 h.

Colonies exhibiting normal *Salmonella* morphology were periodically confirmed by lysine iron agar (Difco), triple sugar iron agar (Difco) slants, and an agglutination assay using *Salmonella* O Poly A-I antiserum (Difco). Samples that were negative from direct plating but positive after RV enrichment were assigned a value of 1.50 log_10_ *Salmonella*/g of cecal contents [43]. Crop samples with less than 0.05 g contents were removed from the study.

### 2.10. Statistical Analysis

Statistical analyses were conducted via Student’s *t*-test. The mean and SEM were calculated for all treatments. Outliers were removed two standard deviations from the mean. All analyses were considered significant if the *p*-value < 0.05.

## 3. Results and Discussion

### 3.1. Biofilm

**Figure 1:**
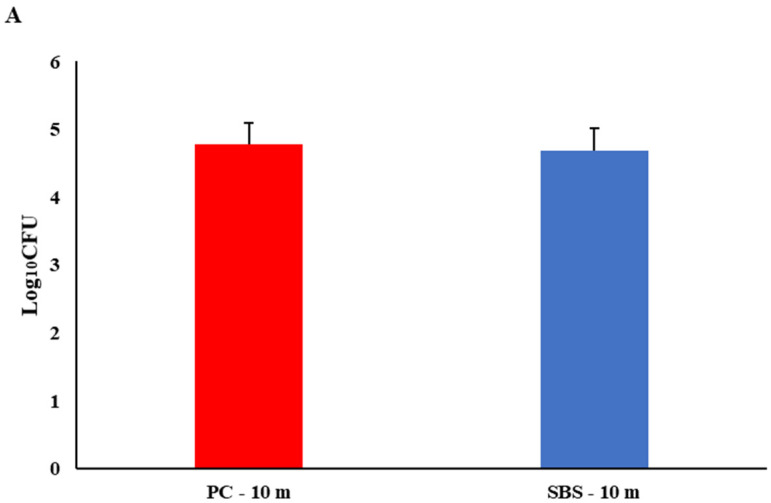

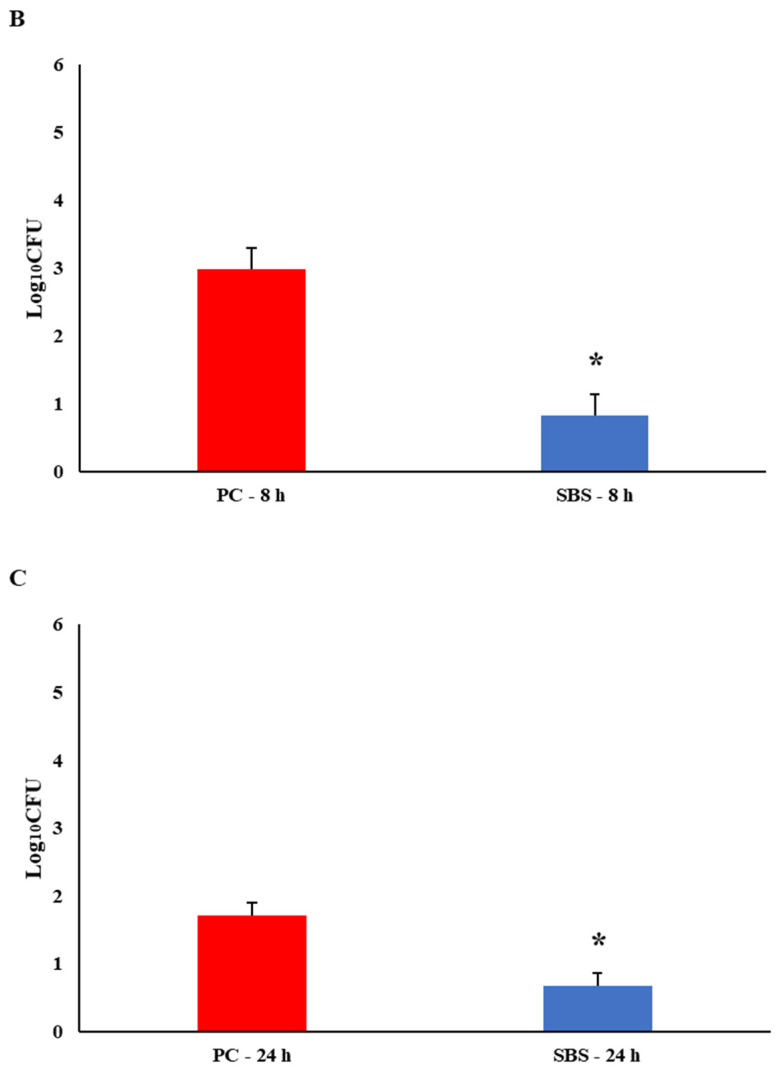
*Salmonella* Heidelberg biofilm on PVC coupons (*n* = 18/treatment) were significantly reduced by sodium bisulfate (SBS) at a pH of 3.5 (*p* < 0.05) * when treated for 8 h or 24 h. The positive control was tap water. Values presented are the averages of 6 separate experiments with triplicate coupon samples within each experiment. Coupons were treated for (**A**) 10 min, (**B**) 8 h, or (**C**) 24 h.

Biofilm production is critical to bacterial persistence [44]. *Salmonella* biofilm on processing surfaces is a food industry concern due to the potential cross-contamination of poultry products [19]. Maharjan and colleagues found that even with consistent water line cleaning, microbial residue would fluctuate depending on the time and location of flocks [45]. Ten minutes was not enough contact time for SBS to significantly decrease SH biofilm (Figure 1A). Sodium bisulfate significantly reduced SH biofilms when applied for 8 h (2.15 log cfu/mL) and 24 h (1.05 log cfu/mL; Figure 1B,C). Overall, SH was a poor biofilm former, which is similar to previous findings [46]. Authors believe SH may have decreased at 24 h due to no supplementation of nutrient medium. Sodium bisulfate could be an efficient and safe way to reduce SH biofilms in poultry drinkers.

### 3.2. Horizontal Transfer

There were no differences in pen weights, feed consumption, or water consumption across treatments, indicating no negative treatment effects of SBS in the water. All incidence of SH in fecal grabs, pre-seeder, and seeder ceca were positive (data not shown).

Acidifying the crop is proactive in bacteria inhibition because it is the second organ in the gastrointestinal tract [47]. Low pH inhibits pathogens, such as *Salmonella*, by acidifying the cell cytoplasm [48]. Ricke reviewed the importance of analyzing the crop in the initial colonization stages of *Salmonella* Enteritidis [49]. Crop colonization was significantly decreased (*p* < 0.05) in the SBS-treated group in trials 1 and 2 (Table 1 and Table 2). Crop pH was significantly decreased (*p* < 0.05) in the SBS-treated group in trial 2 (Table 2). The broiler crop pH can range from below five to greater than six, due to the fermentation of feed by host lactobacilli, which produce lactic acid [50,51,52,53]. The acid-binding (buffering) capacity of feed ingredients can also affect crop pH [51]. The crop contains 10^8^ to 10^9^ cfu/g of primarily Gram-positive facultative anaerobic bacteria such as *Lactobacillus* [48].

Homeostatic pH values are maintained through the bicarbonate cycle [54]. Hinton and colleagues reported the average pH of market-age broiler ceca was 6.2 [55]. Cecal colonization was significantly decreased (*p* < 0.05) in trial 1 (Table 1). In trial 2, there was no significant reduction in SH in cecal colonization (Table 2). We believe this is due to the bicarbonate cycle maintaining homeostatic pH. Other applications of SBS did not see reductions. Harris and colleagues found that *Salmonella* Typhimurium was not significantly decreased by SBS in water in crops and ceca (direct plating and enrichment) of market-age broilers during feed/water withdrawal [56]. Cochrane and colleagues treated ST-contaminated feed ingredients (feather meal, avian blood meal, porcine meat, and bone meal, and poultry by product meal) with 1.0% SBS over a 42-day period and did not see a reduction compared to the control [57]. Line and Bailey applied SBS to broiler houses before chicks were placed and on week 4, no significant effect on *Salmonella* prevalence was detected in fecal grabs and drag swabs [58]. When SBS (4.5 kg/t to 9 kg/t) was added to feed, it did not reduce SE in 34 d post-infected broiler cecas, feces, spleens, or livers when challenged with 2 × 10^5^ cfu/mL on d 1 [59].

We did not withdraw feed, due to the age of the birds and the unlikeliness of the scenario for chicks during brood. Reports have demonstrated that *Salmonella* increases in crops after feed withdrawal (10% versus 1.9%) [60]. Researchers speculate that this is caused by consumption of contaminated litter by the birds during the withdrawal period, because birds continuously peck and consume excreta in litter [47]. Previous experiments with SBS at a pH of 3.2 in drinking water did not impact *Salmonella* Typhimurium in market-age broiler crops or ceca during feed withdrawal [56].

The pH of the water fluctuated during the first 48 h, when the contact and seeder birds were placed in trial 1 due to uncovered drinkers. The drinkers also leaked, which caused a damp and humid environment for the SH to thrive. For trial 2, the drinkers were replaced, which led to dry litter. Garden sprayers were used to add 7 L of water per pen. Interestingly, we saw more differences in SH in the first trial’s results possibly due to the increased water activity.

Water treatments are important to reduce pathogens on farms [61]. Prevention of *Salmonella* in water lines can also reduce cross-contamination during production [21]. Pope and Cherry reported SBS used as an antimicrobial agent and litter acidifier reduced the prevalence of *E. coli* and *Salmonella* in broiler houses [33]. Payne and colleagues reported adjusting turkey litter to a pH of 4.0, with hydrochloric acid being effective in reducing *Salmonella* populations [62]. Inhibition of pH-sensitive pathogenic bacteria, such as *Salmonella*, can occur with the application of acidifiers at a pH below 5 [63]. The use of SBS as a water acidifier to decrease bacterial incidence would be less expensive than organic acids [39].

## 4. Conclusions

Biofilms and planktonic bacteria respond differently to antimicrobial agents, so the goal of this study was to analyze the differences in zoonotic SH versus as a mono-species biofilm. Sodium bisulfate at a pH of 3.5 was able to reduce *Salmonella* Heidelberg biofilm ceca and crop colonization in chicks. Reducing SH in the gut could prevent the fecal contamination of poultry meat during production; however, future trials would be required to determine this. Effective *Salmonella* control will involve multiple intervention strategies. The use of acidifiers in poultry production is one of many tools available that may be used to improve biosecurity and food safety.

## Figures and Tables

**Table 1:** Trial 1 colonization and incidence of crop and cecal contents and average crop pH.

Treatment	Mean Log_10_ cfu/mL ^2^		Enrichment		Average Crop pH ^3^
	Crop	Ceca	Crop	Ceca	
**Positive Control**	3.40 ± 0.04	4.06 ± 0.11	90/90	90/90	5.30 ± 0.07
**SBS**	**3.13 ± 0.86 ^1^**	**3.67 ± 0.12 ^1^**	90/90	90/90	5.24 ± 0.07

^1^*p* < 0.05, statistically significant values were bolded. ^2^ Values are mean ± SEM from 10 birds per pen per treatment with 9 replicate pens. ^3^ Crops of 5 birds per pen were collected for pH measurements. SBS, sodium bisulfate.

**Table 2:** Trial 2 colonization and incidence of crop and cecal contents and average crop pH.

Treatment	Mean Log cfu/mL ^2^		Enrichment		Average Crop pH ^3^
	Crop	Ceca	Crop	Ceca	
**Positive Control**	1.13 ± 0.11	2.01 ± 0.13	55/85	77/90	5.50 ± 0.09
**SBS ^1^**	**0.74 ± 0.01 ^1^**	2.00 ± 0.14	48/83	72/90	**5.05 ± 0.13 ^1^**

^1^*p* < 0.05, statistically significant values were bolded. ^2^ Values are mean ± SEM from 10 birds per pen per treatment with 9 replicate pens. ^3^ Crops of 5 birds per pen were collected for pH measurements. SBS, sodium bisulfate.
